# Pre-Conditioning with CDP-Choline Attenuates Oxidative Stress-Induced Cardiac Myocyte Death in a Hypoxia/Reperfusion Model

**DOI:** 10.1155/2014/187071

**Published:** 2014-01-21

**Authors:** Héctor González-Pacheco, Aurelio Méndez-Domínguez, Salomón Hernández, Rebeca López-Marure, Maria J. Vazquez-Mellado, Cecilia Aguilar, Leticia Rocha-Zavaleta

**Affiliations:** ^1^Departamento de Urgencias y Unidad Coronaria, Instituto Nacional de Cardiología “Ignacio Chavez”, Juan Badiano No. 1, Colonia Seccion 16, 14080 Tlalpan, DF, Mexico; ^2^Departamento de Neurologia, Instituto Nacional de Cardiología “Ignacio Chavez”, Juan Badiano No. 1, Colonia Seccion 16, 14080 Tlalpan, DF, Mexico; ^3^Facultad de Medicina, Universidad Panamericana, Augusto Rodin 498, 03920 Insurgentes Mixcoac, DF, Mexico; ^4^Departamento de Biologia Celular, Instituto Nacional de Cardiología “Ignacio Chavez”, Juan Badiano No. 1, Colonia Seccion 16, 14080 Tlalpan, DF, Mexico; ^5^Departamento de Biología Molecular y Biotecnología, Instituto de Investigaciones Biomédicas, UNAM, Circuito Escolar s/n, Ciudad Universitaria, 04510 Coyoacan, DF, Mexico

## Abstract

*Background*. CDP-choline is a key intermediate in the biosynthesis of phosphatidylcholine, which is an essential component of cellular membranes, and a cell signalling mediator. CDP-choline has been used for the treatment of cerebral ischaemia, showing beneficial effects. However, its potential benefit for the treatment of myocardial ischaemia has not been explored yet. *Aim*. In the present work, we aimed to evaluate the potential use of CDP-choline as a cardioprotector in an *in vitro* model of ischaemia/reperfusion injury. *Methods*. Neonatal rat cardiac myocytes were isolated and subjected to hypoxia/reperfusion using the coverslip hypoxia model. To evaluate the effect of CDP-choline on oxidative stress-induced reperfusion injury, the cells were incubated with H_2_O_2_ during reperfusion. The effect of CDP-choline pre- and postconditioning was evaluated using the cell viability MTT assay, and the proportion of apoptotic and necrotic cells was analyzed using the Annexin V determination by flow cytometry. *Results*. Pre- and postconditioning with 50 mg/mL of CDP-choline induced a significant reduction of cells undergoing apoptosis after hypoxia/reperfusion. Preconditioning with CDP-choline attenuated postreperfusion cell death induced by oxidative stress. *Conclusion*. CDP-choline administration reduces cell apoptosis induced by oxidative stress after hypoxia/reperfusion of cardiac myocytes. Thus, it has a potential as cardioprotector in ischaemia/reperfusion-injured cardiomyocytes.

## 1. Introduction

CDP-choline (cytidine-5-diphosphocholine) is a mononucleotide produced in all mammalian cells. CDP-choline is a key intermediate in the *de novo* biosynthesis of phosphatidylcholine (PC), which is an essential component of cellular membranes, a major brain phospholipid, a choline donor for the synthesis of the neurotransmitter acetylcholine, and also a cell signalling mediator [1]. Exogenously administered CDP-choline known as citicoline is hydrolysed to cytidine and choline by membrane-associated phosphodiesterases. These metabolites mediate the pharmacological and physiological effects attributed to CDP-choline, and also contribute to the resynthesis of CDP-choline in damaged cells [2]. CDP-choline has been used for the treatment of traumatic brain injury [3] and cerebral ischaemia [4], showing beneficial effects, good tolerance, and rare side effects [5, 6].

Although ischaemia can be experimented by any tissue suffering from a restriction in blood supply, benefits of CDP-choline administration have only been studied in the cerebral ischaemic condition. Interestingly, in a pioneer work, Choy and colleagues observed a significant reduction in the rates of PC biosynthesis in hypoxic and ischaemic hamster hearts, mainly caused by a decreased conversion of phosphocholine to CDP-choline [7], suggesting that CDP-choline may be beneficial in the compromised heart [2].

Myocyte damage during cardiac ischaemia occurs through a number of events. After the onset of focal ischaemia, cells in the central area of severe blood flow deficit die rapidly. However, in peripheral areas with a moderate blood flow deficiency, damaged cells remain viable and can be rescued with a timely intervention. On the other hand, reperfusion may also cause cell death by molecular mechanisms including inflammation and oxidative stress [8].

Ischaemia/reperfusion injury is mediated by elements secreted by both, injured cardiomyocytes and inflammatory cells. Endogenous reactive oxygen species (ROS) are produced by harmed mitochondria in ischaemia/reperfusion-injured cells [9]. In addition, inflammatory cells, neutrophils in particular, are the main source of exogenous ROS after reperfusion [10]. Furthermore, inflammatory cells also secrete a number of toxic cytokines, such as interleukin 1*β* (IL-1*β*), IL-8, and tumor necrosis factor-*α* (TNF-*α*). TNF-*α* is a strong inducer of apoptosis and necrosis in myocytes [11]. Therefore, in the present work, we aimed to evaluate the potential use of CDP-choline either as a preconditioner or postconditioner in an *in vitro* model of hypoxia/reperfusion injury using isolated myocardial cells.

## 2. Materials and Methods

### 2.1. Neonatal Cardiac Myocyte Culture

Male, 1–4-day-old Wistar rats were provided by the Animal House, Faculty of Medicine, Panamerican University. Experimental procedures were carried out in accordance with local and international guidelines for care and use of laboratory animals. Rats were anaesthetized by an intraperitoneal injection of ketamine : xylazine (75 : 10 mg/kg); neonatal cardiomyocytes were isolated from rat ventricles by digestion with 0.7% trypsin (Sigma Chemical Company, St. Louis, MO, USA) overnight at 4°C, followed by digestion with 2 mg/mL type 2 collagenase (Sigma Chemical Company, St. Louis, MO, USA) for 2 hours (hrs) at 37°C with gentle shaking. Cells were grown in Leibovitz L-15 medium (*In Vitro*, Mexico, DF, Mexico) supplemented with 10% Foetal Calf Serum, in a humidified incubator at 37°C under 5% CO_2_/air.

### 2.2. Hypoxia/Reperfusion Model

Hypoxia was induced using the coverslip hypoxia model [12] with some modifications. 1 × 10^6^ neonatal cardiac myocytes were plated onto 35 mm dishes and incubated as described above for 24 hrs. The cells were covered using round, 18 mm diameter coverslips and incubated in glucose-free/serum-free Leibovitz L-15 medium for 15, 30, 60, 120, and 180 minutes (min). After the specified time, the coverslips were removed using forceps. Cell viability was calculated by using the colorimetric MTT assay. For reperfusion events, the coverslips were removed, and the glucose-free/serum-free medium was replaced by complete medium; the cells were then incubated as described above.

### 2.3. CDP-Choline Cardioprotection Assays

To evaluate the effect of CDP-choline on cardiac myocytes, 4 × 10^4^ cells were plated onto 96-well plates and incubated in the presence of 0.5, 1, 2.5, 5, 10, 20, 30, 40, 50, 75, and 100 mg/mL of CDP-choline (Ferrer Internacional, S.A., Barcelona, Spain) for 24 and 48 hrs. The number of cells was determined by using the colorimetric MTT assay. For cardioprotection assays, CDP-choline (10, 25, and 50 mg/mL) was added either 1 hr before (preconditioning) or 1 hr after (postconditioning) reperfusion. The number of viable cells was determined after 24 hrs. To assess changes in cell morphology, cells were washed twice with cold PBS and fixed in 100% ethanol overnight at room temperature. The remaining ethanol was discarded, and the cells were rehydrated with double distilled water for 5 min. The cells were stained with hematoxylin and eosin (Biocare Medical, CA, USA). Stained cells were analyzed using a light microscopy Axiostar Plus (Karl Zeiss Microscopy GmbH, Jena, Germany). To evaluate the effect of CDP-choline on oxidative stress-induced death, 5 mM H_2_O_2_ (Sigma Chemical Company, St. Louis, MO, USA) was added immediately after reperfusion, and the cells were further incubated for 6 hrs.

### 2.4. Western Blot Analysis

The cells were harvested and resuspended in lysis buffer (50 mM Tris-HCl, pH 7.4; 150 mM NaCl; 1 mM EDTA; 1% NP40; 0.25% sodium deoxycholate) containing 100 *μ*L/mL of Complete Protease Inhibitors Cocktail (Roche Applied Science, Mannheim, Germany) and 10 *μ*L/mL of phosphatases inhibitor (Sigma-Aldrich, Saint Louis, MS). Total protein content was determined using the DC Protein Assay Kit (BioRad Laboratories, Hercules, CA). 40 *μ*g of protein was resolved by electrophoresis on a vertical 10% SDS-PAGE and transferred to polyvinylidene fluoride (PVDF) membranes (Millipore, Billerica, MA, USA). Membranes were incubated with primary antibodies overnight at 4°C, washed and incubated with the appropriate horseradish peroxidase-conjugated secondary antibody (Zymed Laboratories, Invitrogen, Carlsbad, CA, USA). Proteins were detected by using the Immobilon Western Chemiluminescent HRP Substrate (Millipore, Billerica, MA, USA). The antibodies used were rabbit monoclonal anti-HIF-1*α* (GeneTex Inc., Irvine, CA, USA) diluted at 1 : 5000 and HRP-conjugated goat anti-rabbit IgG (Zymed Laboratories., Invitrogen Co., USA) diluted at 1 : 5000. As an internal control, a rabbit anti-B-Actin (GeneTex Inc.) was included.

### 2.5. Detection of Apoptosis by Fluorescence Microscopy

To assess nuclear morphology changes associated with apoptosis, the cells were grown onto glass slides, the culture media were removed, and the cells were fixed in 3.7% buffered formaldehyde for 10 min at room temperature. After incubation in phosphate buffered saline (PBS) for 10 min, the slides were washed with deionised water and stained with Hoechst 33342 fluorescent dye (Thermo Fisher Scientific Inc. Rockford, IL, USA) diluted at 1 *μ*g/mL for 15 min at room temperature. The slides were washed and mounted. The cells were analyzed using a Fluorescence Microscope Axiostar Plus (Karl Zeiss Microscopy GmbH, Jena, Germany).

### 2.6. Quantification of Apoptotic and Necrotic Cells by Flow Cytometry

Apoptotic and necrotic cells were evaluated using the Annexin-V-FLUOS Staining Kit (Roche Diagnostics GmbH, Mannheim, Germany). The cells were washed with PBS and centrifuged at 200 ×g for 5 min. Cell pellets were resuspended in 100 *μ*L of labelling solution, containing 20 *μ*L Annexin-V-FLUOS labelling reagent and 1 *μ*g/mL propidium iodide and incubated for 15 min at room temperature. The samples were analyzed by flow cytometry using a FACSCalibur (Becton Dickinson, San Jose, CA, USA) at 488 nm (excitation) and 515 nm bandpass filter for fluorescein detection and a filter >560 nm for propidium iodide detection. Cells stained with Annexin-V-FLUOS alone were considered apoptotic, whereas double stained cells (Annexin-V-FLUOS + propidium iodide) were considered late apoptotic or necrotic. Data analysis was done using the Cell Quest Software Program.

### 2.7. Statistical Analysis

Results are presented as the mean ± standard error of the mean (SEM). Differences between treatment and control groups were analyzed using the ANOVA test and Tukey-Kramer posttest. Ninety-five percent confidence intervals (CI) and *P* values were calculated. The tests considered a basic significance level of *P* ≤ 0.05.

## 3. Results

### 3.1. Coverslip Hypoxia Model Induces Cardiac Myocyte Death and Expression of HIF-1*α*

**Figure 1:**
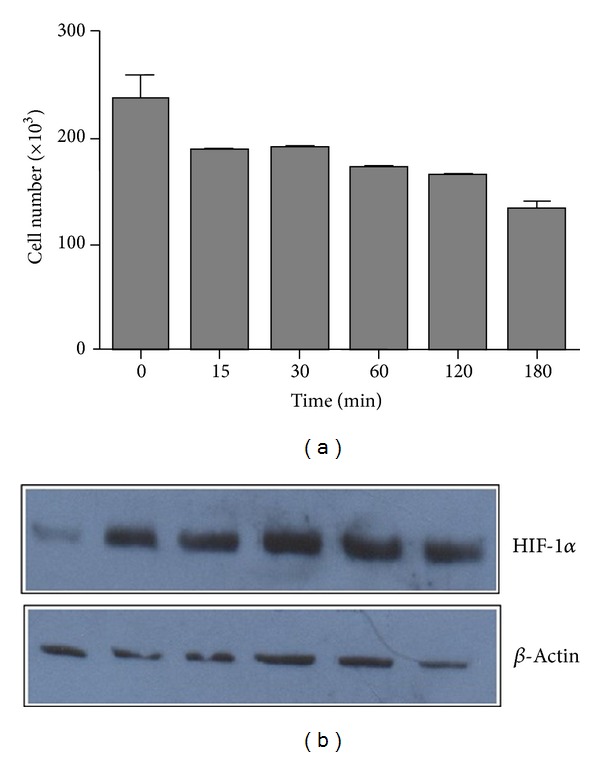
Induction of cell death by the coverslip hypoxia model. (a) Coverslips were placed onto confluent cardiac myocyte monolayers and removed after 15, 30, 60, 120, and 180 min. As a negative control, myocyte cultures were left uncovered. Cell viability was evaluated by the MTT assay. Data represent the average of three independent tests. Error bars indicate the standard error of the mean. (b) Proteins in whole cell lysates obtained at the indicated time points were analyzed for HIF-1*α* expression by immunoblotting. As a control, the expression of the constitutive *β*-actin protein was evaluated.

To corroborate that the coverslip model induces hypoxia-mediated cell death, cardiac myocytes were coverslipped for 15, 30, 60, 120, and 180 min. Cell viability was then evaluated. As seen in Figure 1(a), the number of viable cells decreased in a time-dependent manner. After 180 min, only 57% of cardiac myocytes remained viable. It has been reported that cardiac cells death, under the coverslip, is primarily mediated by hypoxia [12]. In addition, it is known that low intracellular oxygen levels induce the expression of the hypoxia inducible factor-1 alpha (HIF-1*α*). Thus, to determine whether coverslipped myocytes were suffering from intracellular hypoxia, we evaluated the expression of HIF-1*α* in whole cell lysates obtained at the indicated time points. As shown in Figure 1(b), a significant increase of HIF-1*α* was observed after 15 min of hypoxia. The expression of HIF-1*α* correlated with the time course of the coverslip procedure. This observation indicates that myocytes under the coverslip undergo intracellular hypoxia.

### 3.2. Pre- and Postconditioning with CDP-Choline Reduce Hypoxia/Reperfusion-Induced Cell Death

**Figure 2:**
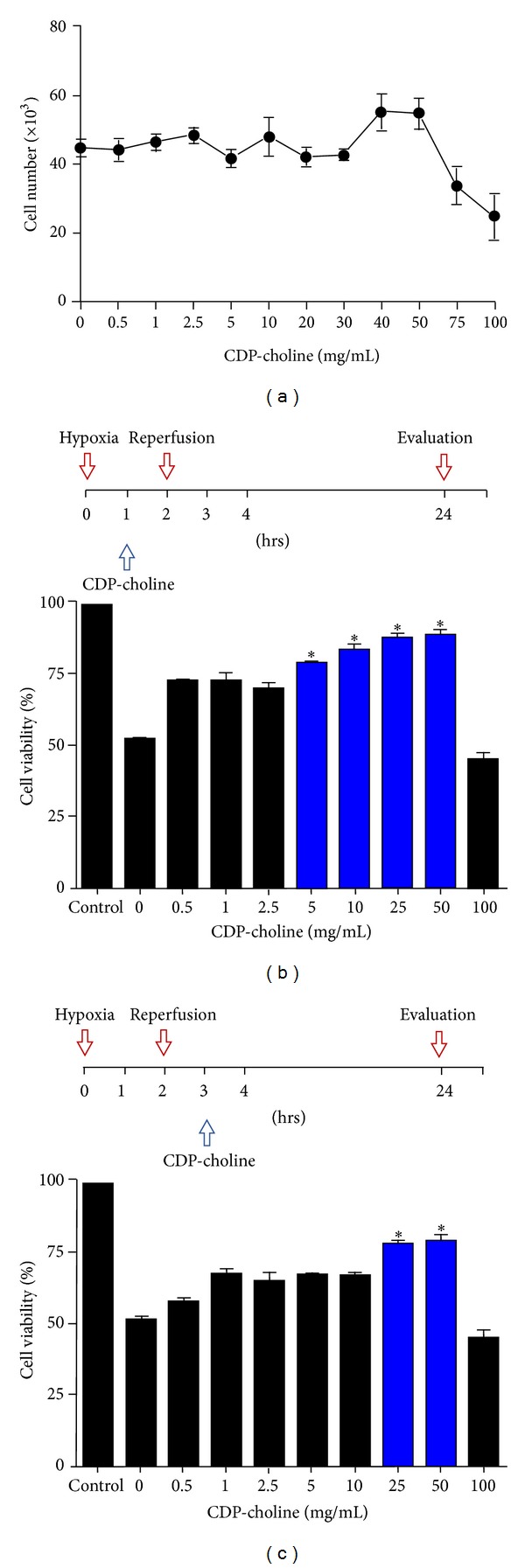
Effect of CDP-choline on cardiac myocytes survival. (a) Cardiac myocyte cultures were treated with increasing concentrations of CDP-choline as indicated. The proportion of viable cells was determined at 24 hrs using the MTT assay. (b) Effect of CDP-choline preconditioning. Cardiac myocytes were coverslipped for 2 hrs. The coverslip was then removed, and the cells were reperfused. CDP-choline was added to coverslipped cells 1 hr prior to reperfusion at the indicated concentrations. Cell viability was assessed at 24 hrs using the MTT assay. (c) Effect of CDP-choline postconditioning. Cardiac myocytes were coverslipped for 2 hrs. The coverslip was then removed, and the cells were reperfused. CDP-choline was added 1 hr after reperfusion. The proportion of viable cells was assessed at 24 hrs using the MTT assay. All values reported represent the average of three independent tests. Error bars indicate the standard error of the mean. *P < 0.05 versus untreated control values.

CDP-choline has been probed to improve functional recovery of neurons after experimental stroke and cerebral ischaemia [13]. Thus, we wanted to investigate whether CDP-choline may attenuate hypoxia/reperfusion-induced cardiac myocyte death. We first evaluated the effect of increasing concentrations of CDP-choline on the viability of cardiac myocytes cultures. As shown in Figure 2(a), administration of 0.5–50 mg/mL of CDP-choline to cell cultures for 24 hrs had no effect on cell viability. However, 75 and 100 mg/mL of CDP-choline induced a significant decrement in the number of cells after 24 hrs, suggesting that CDP-choline may be toxic to the cells at these concentrations. Next, we evaluated the potential protective effect of preconditioning with increasing concentrations of CDP-choline; results are shown in Figure 2(b). Preincubating coverslipped cells with CDP-choline, 1 hr before reperfusion, induced a significant increment of cell viability from 5 up to 50 mg/mL (*P* < 0.05). As expected, preincubation with 100 mg/mL of CDP-choline resulted in a higher level of cell death. This observation suggests that preconditioning with CDP-choline has a positive impact on cell survival after hypoxia/reperfusion. We were further interested in determining the effect of postconditioning with CDP-choline. To this end, coverslipped cells were subjected to reperfusion, and 1 hr later they received increasing concentrations of CDP-choline. Results are depicted in Figure 2(c). A significant increase of cell viability was detected at concentrations of 25 and 50 mg/mL of CDP-choline (*P* < 0.05). In contrast, the number of viable cells after postconditioning with 100 mg/mL of CDP-choline was not significantly different than that observed in the untreated negative control. These results indicate that postconditioning with CDP-choline protects cardiac myocyte from hypoxia/reperfusion-mediated death.

### 3.3. CDP-Choline Reduces Postreperfusion Cardiac Myocyte Apoptosis but Not Necrosis

**Figure 3:**
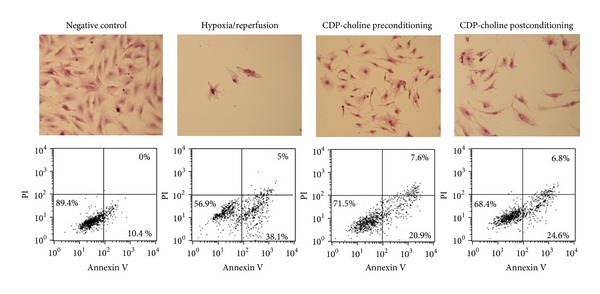
CDP-choline attenuates hypoxia/reperfusion-induced apoptosis. Cardiac myocyte cultures were subjected to coverslip hypoxia/reperfusion alone (Hypoxia/reperfusion), incubated with CDP-choline (50 mg/mL) prior to reperfusion (CDP-choline preconditioning), or incubated with CDP-choline (50 mg/mL) 1 hr after reperfusion (CDP-choline postconditioning). Cell cultures were left untreated as a control (Negative control). After treatment, the cells were stained with hematoxylin-eosin; cell morphology was assessed by light microscopy (40X) (upper panel). Apoptosis and necrosis were evaluated by flow cytometry analysis of cells labeled with Annexin-V-FLUOS (Annexin V) and propidium iodide (PI) (lower panel). The proportion of labeled cells is depicted in the lower panel. The proportion of viable cells, with low Annexin and low PI staining, is shown in the left, lower quadrant of each dot plot. Apoptotic cells, with high Annexin and low PI staining, are depicted in the right, lower quadrant. Necrotic cells, with high Annexin and high PI staining, are showed in the right, upper quadrant. Results shown are representative figures from three independent assays.

Our results suggest that CDP-choline attenuates postreperfusion cardiomyocytes cell death. Hematoxylin-eosin staining of cells showed a clear change in morphology after hypoxia/reperfusion, including extensive cytosolic vacuolization and presence of irregular cell membrane buds (Figure 3, upper panel). The proportion of cells showing this type of morphological changes was smaller in CDP-choline pre- and postconditioned cultures. It is known that the continuous loss of cardiac cells after reperfusion is mainly caused by necrosis and apoptosis. Thus, to evaluate the potential effect of CDP-choline on apoptosis and necrosis, we determined the proportion of viable, apoptotic, and necrotic cells after reperfusion by staining the cells with Annexin V and propidium iodide. Flow cytometry analysis showed that the proportion of apoptotic cells increased significantly after reperfusion from 10.4% in the untreated control to 38.1% in the cells undergoing hypoxia/reperfusion (Figure 3, lower panel). CDP-choline pre- and post-conditioning induced a significant reduction of apoptotic cells (20.9% and 24.6%, respectively). In contrast, the proportion of necrotic cells detected after reperfusion (5.0%) remained unchanged regardless of the presence of CDP-choline. These observations indicate that CDP-choline protective effect is associated with a reduction of apoptosis.

### 3.4. CDP-Choline Protects Cardiac Myocytes from Oxidative Stress-Mediated Postreperfusion Death

**Figure 4:**
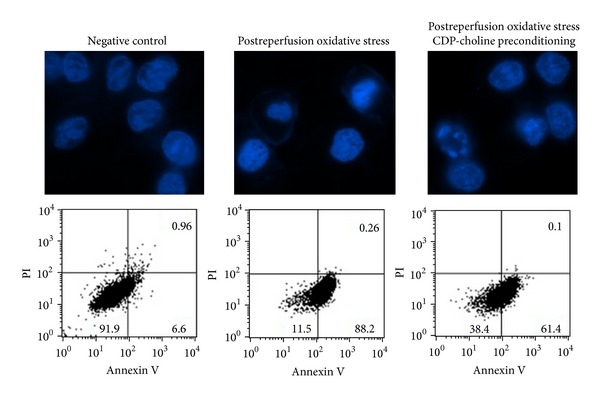
CDP-choline attenuates oxidative stress-induced postreperfusion apoptosis. Cardiac myocyte cultures were subjected to coverslip hypoxia/reperfusion. To induce oxidative stress, 5 mM H_2_O_2_ was added immediately after reperfusion (Postreperfusion oxidative stress). Preconditioning was performed by incubating the cells with CDP-choline (50 mg/mL) prior to reperfusion (Postreperfusion oxidative stress CDP-choline preconditioning). Cells were further incubated for 6 hrs. A group of untreated cells was included as control (negative control). To assess changes in cell nucleus morphology, the cells were fixed and stained with Hoechst 33342 fluorescent dye (upper panel). Changes in nuclear morphology associated with apoptosis, such as nuclear condensation and fragmentation, were detected by fluorescence microscopy (63X). Apoptosis and necrosis were evaluated by flow cytometry analysis of cells labeled with Annexin-V-FLUOS (Annexin V) and propidium iodide (PI) (lower panel). The proportion of labeled cells is depicted in the lower panel. The proportion of viable cells, with low Annexin and low PI staining, is shown in the left, lower quadrant of each dot plot. Apoptotic cells, with high Annexin and low PI staining, are depicted in the right, lower quadrant. Necrotic cells, with high Annexin and high PI staining, are showed in the right, upper quadrant. Results shown are representative figures from three independent assays.

There is strong evidence showing that potent reactive oxygen species (ROS) produced during early stages of reperfusion are associated with reperfusion cell injury [14–16]. Thus, in order to determine the potential effect of CDP-choline on extracellular ROS-induced cell death, myocytes undergoing hypoxia/reperfusion were either preconditioned with CDP-choline or left untreated and exposed to oxidative stress by addition of H_2_O_2_ immediately after reperfusion. Results are shown in Figure 4. Analysis of cells stained with the Hoechst dye demonstrated the presence of nuclear morphology changes associated with apoptosis, such as nuclear condensation and fragmentation, in the cells exposed to oxidative stress. A lower proportion of CDP-choline preconditioned cells showed changes associated with apoptosis (Figure 4 upper panel). Flow cytometry analysis (Figure 4 lower panel) showed a highly significant increase in apoptosis when the cells were exposed to oxidative stress (*P* < 0.005). In fact, the proportion of apoptotic cells augmented from 6.6% in the untreated negative control to 88.2% in the oxidative stress group. Preincubation of cells with CDP-choline induced a significant reduction in apoptosis, resulting in a proportion of 61.4% apoptotic cells.

## 4. Discussion

Acute myocardial infarction remains a leading cause of death all over the world. Therefore, there is a strong need for new strategies for cardioprotection. Here, we investigated the potential cardioprotective effect of CDP-choline. To that end, we used the coverslip hypoxia model on neonatal rat cardiomyocytes, which mimics the formation of transition regions between a hypoxic-ischaemic zone and a nonischaemic zone. To demonstrate that coverslipped cells were actually undergoing hypoxia, we evaluated the expression of HIF-1*α*. Our results showed an increase of HIF-1*α* expression during the induction of hypoxia. Since expression and stabilization of HIF-1*α* is considered the hallmark of the cellular response to hypoxia, our results confirm that the coverslip model reproduces the hypoxic event in cardiac myocytes.

Previous studies revealed that hypoxia/reperfusion induces apoptosis in the human [17] and rat heart [18]. Accordingly, in this work we observed a high proportion of cardiac myocytes undergoing apoptosis after reperfusion. Interestingly, pre-conditioning and post-conditioning with CDP-choline attenuated postreperfusion cardiac myocyte apoptosis but had no impact on cell necrosis. Apoptosis in the ischaemic-reperfused heart is mediated by a complex network of signals released by the injured cardiomyocyte itself and by cells of the inflammatory response. A crucial element mediating cardiomyocytes apoptosis is oxidative stress. Injured myocytes show increased concentrations of mitochondria-derived ROS [19]. Moreover, activated inflammatory cells secrete considerable amounts of ROS to the extracellular microenvironment [19]. Here, we found that pre-conditioning with CDP-choline induced a significant reduction of apoptosis produced by oxidative stress to reperfused cardiomyocytes.

Cell death after ischaemia/reperfusion is mediated by necrosis and apoptosis. However, ROS have been shown to directly mediate apoptosis after reperfusion injury [20]. ROS causes denaturation of proteins, such as enzymes and ion channels, and peroxidation of membrane lipids, which in turn may affect membrane integrity. A careful analysis of the distribution of cell membrane phospholipids showed a significant increase in the content of lysophosphatidylcholine (LPC) in cardiomyocytes after ischaemia/reperfusion [21]. LPC is a product of lipid peroxidation. Therefore, accumulation of LPC during ROS-induced reperfusion injury is expected. LPC has been demonstrated to be a potent inducer of apoptosis in various types of cells, including endothelial vascular cells [22] and hepatocytes [23]. Moreover, it has been demonstrated that small changes in the LPC/PC ratio may produce neuronal cell death [24]. Here, we found that addition of CDP-choline attenuates ROS-mediated postreperfusion cell death. This effect may be associated with the fact that CDP-choline reduces the level of LPC and increases the activity of CTP: phosphocholine cytidylyltransferase [25], which is considered to be the rate-limiting enzyme of the pathway for the synthesis of PC [26]. However, further investigation to ascertain the mechanism leading to the effect detected herein will be needed.

PC is not only a major component of cell membranes, but also a cell signalling mediator. Thus, preserving PC homeostasis may have a positive impact on the survival of cells after a hypoxia/reperfusion insult. CDP-choline has been found to be of benefit in ischaemia and hypoxia in stroke and other central nervous system disorders [27]. However, the effects of CDP-choline on cardiac ischaemia have been less investigated. In 1992, Neidhardt and colleagues [28] demonstrated for the first time that CDP-choline is able to protect rat myocardial cells against the detrimental effect of anoxia on heart rate and force of contraction (inotropism), suggesting that CDP-choline may be useful in the treatment of myocardial ischaemia. In line with that pioneer work, here we have presented evidence that CDP-choline attenuates cell death induced by hypoxia-reperfusion, and, in particular, we have shown that this effect may be associated with protection against ROS-induced apoptosis. Therefore, our results support the idea that CDP-choline has a potential as a cardioprotector. However, to demonstrate that CDP-choline can protect against myocardial ischaemia, a protocol using intact hearts is guaranteed.

## References

[1] Exton JH (1994). Phosphatidylcholine breakdown and signal transduction. *Biochimica et Biophysica Acta*.

[2] Weiss GB (1995). Metabolism and actions of CDP-choline as an endogenous compound and administered exogenously as citicoline. *Life Sciences*.

[3] Zafonte R, Friedewald WT, Lee SM (2009). The citicoline brain injury treatment (COBRIT) trial: design and methods. *Journal of Neurotrauma*.

[4] Ortega G, Jacas C, Quintana M (2010). Citicoline treatment prevents neurocognitive decline after a first ischemic stroke. *Cerebrovascular Diseases*.

[5] Saver JL (2008). Citicoline: update on a promising and widely available agent for neuroprotection and neurorepair. *Reviews in Neurological Diseases*.

[6] Cho HJ, Kim YJ (2009). Efficacy and safety of oral citicoline in acute ischemic stroke: drug surveillance study in 4,191 cases. *Methods and Findings in Experimental and Clinical Pharmacology*.

[7] Choy PC, Chan M, Hatch G, Man RYK (1992). Phosphatidylcholine metabolism in ischemic and hypoxic hearts. *Molecular and Cellular Biochemistry*.

[8] Baines CP (2011). How and when do myocytes die during ischemia and reperfusion: the late phase. *Journal of Cardiovascular Pharmacology and Therapeutics*.

[9] Vanden Hoek TL, Li C, Shao Z, Schumacker PT, Becker LB (1997). Significant levels of oxidants are generated by isolated cardiomyocytes during ischemia prior to reperfusion. *Journal of Molecular and Cellular Cardiology*.

[10] Vinten-Johansen J (2004). Involvement of neutrophils in the pathogenesis of lethal myocardial reperfusion injury. *Cardiovascular Research*.

[11] Haudek SB, Taffet GE, Schneider MD, Mann DL (2007). TNF provokes cardiomyocyte apoptosis and cardiac remodeling through activation of multiple cell death pathways. *Journal of Clinical Investigation*.

[12] Pitts KR, Toombs CF (2004). Coverslip hypoxia: a novel method for studying cardiac myocyte hypoxia and ischemia *in vitro*. *The American Journal of Physiology—Heart and Circulatory Physiology*.

[13] Hurtado O, Cárdenas A, Pradillo JM (2007). A chronic treatment with CDP-choline improves functional recovery and increases neuronal plasticity after experimental stroke. *Neurobiology of Disease*.

[14] Bolli R, Jeroudi MO, Patel BS (1989). Direct evidence that oxygen-derived free radicals contribute to postischemic myocardial dysfunction in the intact dog. *Proceedings of the National Academy of Sciences of the United States of America*.

[15] Mergner GW, Weglicki WB, Kramer JH (1991). Postischemic free radical production in the venous blood of the regionally ischemic swine heart. Effect of deferoxamine. *Circulation*.

[16] Grill HP, Zweier JL, Kuppusamy P, Weisfeldt ML, Flaherty JT (1992). Direct measurement of myocardial free radical generation in an *in vivo* model: effects of postischemic reperfusion and treatment with human recombinant superoxide dismutase. *Journal of the American College of Cardiology*.

[17] Itoh G, Tamura J, Suzuki M (1995). DNA fragmentation of human infarcted myocardial cells demonstrated by the nick end labeling method and DNA agarose gel electrophoresis. *The American Journal of Pathology*.

[18] Scarabelli TM, Stephanou A, Pasini E (2002). Different signaling pathways induce apoptosis in endothelial cells and cardiac myocytes during ischemia/reperfusion injury. *Circulation Research*.

[19] Zweier JL, Talukder MAH (2006). The role of oxidants and free radicals in reperfusion injury. *Cardiovascular Research*.

[20] Maulik N, Yoshida T, Das DK (1998). Oxidative stress developed during the reperfusion of ischemic myocardium induces apoptosis. *Free Radical Biology and Medicine*.

[21] Maulik N, Kagan VE, Tyurin VA, Das DK (1998). Redistribution of phosphatidylethanolamine and phosphatidylserine precedes reperfusion-induced apoptosis. *The American Journal of Physiology—Heart and Circulatory Physiology*.

[22] Hsieh CC, Yen MH, Liu HW, Lau YT (2000). Lysophosphatidylcholine induces apoptotic and non-apoptotic death in vascular smooth muscle cells: in comparison with oxidized LDL. *Atherosclerosis*.

[23] Kakisaka K, Cazanave SC, Fingas CD (2012). Mechanisms of lysophosphatidylcholine-induced hepatocyte lipoapoptosis. *The American Journal of Physiology—Gastrointestinal and Liver Physiology*.

[24] Mulder C, Wahlund LO, Teerlink T (2003). Decreased lysophosphatidylcholine/phosphatidylcholine ratio in cerebrospinal fluid in Alzheimer’s disease. *Journal of Neural Transmission*.

[25] Adibhatla RM, Hatcher JF, Dempsey RJ (2004). Cytidine-5′-diphosphocholine affects CTP-phosphocholine cytidylyltransferase and lyso-phosphatidylcholine after transient brain ischemia. *Journal of Neuroscience Research*.

[26] Kent C (1997). CTP:phosphocholine cytidylyltransferase. *Biochimica et Biophysica Acta*.

[27] Adibhatla RM, Hatcher JF (2005). Cytidine 5′-diphosphocholine (CDP-choline) in stroke and other CNS disorders. *Neurochemical Research*.

[28] Neidhardt A, Costes Y, Bachour K, Platonoff N (1992). Effect of cytidine diphosphate choline on anoxia tolerance of cultured myocardial cells. *Clinical Therapeutics*.

